# High Thermoelectric Performance in Solution‐Processed Semicrystalline PEDOT:PSS Films by Strong Acid–Base Treatment: Limitations and Potential

**DOI:** 10.1002/advs.202308368

**Published:** 2024-01-18

**Authors:** Juhyung Park, Jae Gyu Jang, Keehoon Kang, Sung Hyun Kim, Jeonghun Kwak

**Affiliations:** ^1^ Department of Electrical and Computer Engineering Inter‐University Semiconductor Research Center Soft Foundry Institute Seoul National University Seoul 08826 Republic of Korea; ^2^ Department of Carbon Convergence Engineering Wonkwang University Iksan 54538 Republic of Korea; ^3^ Department of Materials Science and Engineering Research Institute of Advanced Materials Institute of Applied Physics Seoul National University Seoul 08826 Republic of Korea

**Keywords:** charge transport, doping, PEDOT:PSS, percolation, polymer thermoelectrics, sequential treatment, tie‐chains

## Abstract

Thermoelectric (TE) generation with solution‐processable conducting polymers offers substantial potential in low‐temperature energy harvesting based on high tunability in materials, processes, and form‐factors. However, manipulating the TE and charge transport properties accompanies structural and energetic disorders, restricting the enhancement of thermoelectric power factor (*PF*). Here, solution‐based strong acid–base treatment techniques are introduced to modulate the doping level of poly(3,4‐ethylenedioxythiophene):poly(styrenesulfonate) (PEDOT:PSS) thin films with preserving its molecular orientation, enabling to achieve a remarkably high *PF* of 534.5 µW m^−1^ K^−2^. Interestingly, theoretical modeling suggested that further de‐doping can increase the *PF* beyond the experimental value. However, it is impossible to reach this value experimentally, even without any degradation of PEDOT crystallinity. Uncovering the underlying reason for the limitation, an analysis of the relationship among the microstructure–thermoelectric performance–charge transport property revealed that inter‐domain connectivity via tie‐chains and the resultant percolation for transport are crucial factors in achieving high TE performance, as in charge transport. It is believed that the methods and fundamental understandings in this work would contribute to the exploitation of conducting polymer‐based low‐temperature energy harvesting.

## Graphical abstract

Optimized strong acid–base treatment is developed to effectively control the doping level of poly(3,4‐ethylendioxythiophene):poly‐(styrenesulfonate) thin films while preserving their microstructure, leading to a remarkably high thermoelectric power factor of 534.5 µW m^−1^ K^−2^. The study further emphasizes the critical role of tie‐chain connectivity and resulting percolation to achieve a theoretical maximum thermoelectric efficiency.

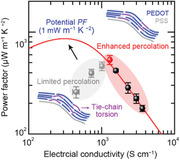

## 1 Introduction

Recently, there has been a growing interest in energy harvesting as an environmentally sustainable energy source to meet the global demands for coping with climate change.^[^ ^1^ ^]^ Among various energy harvesting technologies, thermoelectric (TE) generation, which can directly convert thermal energy into electricity, has been regarded as a promising energy conversion system based on semiconductors, leading to extensive research and commercial production of TE generators (TEGs) based on inorganic semiconductors.^[^ ^2^, ^3^, ^4^ ^]^ However, their low heat‐to‐electricity conversion efficiency from low‐temperature thermal energy that occupies ≈60% of total waste heat restricts the practical use of TEGs in our daily lives. Efficient harvesting of low‐temperature heat resources, such as the human body, would be a promising way of supplying electricity to low‐power wearable devices and Internet of Things sensors.^[^ ^5^ ^–^ ^7^ ^]^

For this purpose, conducting polymers are one of the most suitable materials for low‐temperature TEGs owing to their inherent nature of lightweight, flexibility, and low toxicity, as well as cost‐effective synthesis and solution processability.^[^ ^8^, ^9^, ^10^ ^]^ Also, continuous evolution in material design and doping methods have led to the improvement of polymer film‐based TE performance which is typically expressed as a power factor *PF* = *α* ^2^ *σ*, where *α* and *σ* are the Seebeck coefficient and electrical conductivity, respectively.^[^ ^11^, ^12^, ^13^, ^14^, ^15^, ^16^ ^]^ To maximize the *PF*, fine‐tuning of charge carrier concentration *n* through doping and/or de‐doping with an additive is known as the key strategy because of the interplay between *σ* and *α* with respect to *n*.^[^ ^17^ ^]^ However, complications occur because the doping/de‐doping process can induce a structural perturbation on polymer backbones increasing the energetic disorder.^[^ ^18^, ^19^, ^20^, ^21^ ^]^ This dopant‐induced disorder strongly affects the charge transport in polymer films, and therefore the inter‐relationship among *n*, charge carrier mobility *µ*, and the other TE parameters makes it difficult to predict the maximum *PF* as a function of *n*.^[^ ^22^ ^]^ Thus, as a new guiding principle to find out the optimum *PF* of polymer TE devices, the importance of exploring the relation between *α* and *σ* has continued to grow.^[^ ^23^, ^24^, ^25^ ^]^ So far, a power law of *α* ∝ *σ* ^−1/^ *^s^* has been widely used for describing the empirical *α*–*σ* relationship in conjugated polymers, where *s* is related to describing the energy dependence of transport function in the charge transport edge model. Researchers have empirically shown that several doped semiconducting polymers exhibit the power law with *s* = 3 (or 4). But when the *s* = 3 model predicts a relatively small gain in the *PF* by solely increasing the doping level,^[^ ^26^ ^]^ which posed challenges in significantly optimizing the *PF* without approaching almost unphysically high doping levels.

Based on simulation results, recent studies have suggested that the degree of disorder is correlated with parameter *s*. Therefore, reducing the degree of disorder can potentially lead to an enhanced *PF* by decreasing the parameter *s* (approaching a value of 1).^[^ ^27^, ^28^, ^29^ ^]^ Poly(3,4‐ethylenedioxythiophen):tosylate (PEDOT:Tos) is a representative example of the *s* = 1 system, demonstrating a superior *PF* compared to other doped polymers due to its disorder‐free charge transport characteristics. The semi‐metallic behavior stemming from high crystallinity with degenerate bipolaron states enables the achievement of a high *PF* based on a large *α* at high *σ* values.^[^ ^12^ ^]^ Although a few films prepared by oxidative polymerization of 3,4‐ethylenedioxythiophen (EDOT) monomers (e.g., PEDOT:Tos, and PEDOT synthesized through an oxidative chemical vapor deposition) have shown high *PF* accompanied by the *s* = 1 feature,^[^ ^11^, ^30^ ^]^ these films have limited processibility (e.g., substrate, patterning) and scalability for TEG applications. Thus, as a TE active material, it is advantageous to use water‐dispersed PEDOT stabilized by polystyrene sulfonate (PSS), PEDOT:PSS, which is one of the most well‐established polymeric‐conductors in terms of film deposition and patterning based on conventional solution processes. Moreover, the *σ* of a PEDOT:PSS thin film can be dramatically improved (up to several thousand siemens per centimeters) through various pre‐ and post‐treatments using organic solvents, surfactants, and acids,^[^ ^31^, ^32^ ^]^ allowing the development of high‐performance TEG modules with low output impedance. However, due to their microstructural heterogeneity caused by the phase separation of PEDOT‐rich and PSS‐rich domains (and dopants if added),^[^ ^33^ ^]^ the inter‐relationships between the TE and charge transport properties could not be investigated thoroughly, which is a major obstacle hindering the further improvements of the performance of TE devices using solution‐processible PEDOT:PSS. In other words, it is vital to investigate how the PEDOT:PSS microstructures (including the domain crystallinity and connectivity through tie‐chains) affect the macro‐/micro‐scale charge transport and TE properties, in order to reveal the contributing factors to the *α*–*σ* relationship and the *PF*.

Here we demonstrate a solution‐processed, high‐performance TE device based on a PEDOT:PSS thin film and thoroughly investigate their microstructure–thermoelectric performance–charge transport property relationships. We first fabricated a highly conductive PEDOT:PSS films using a strong acid, trifluoromethanesulfonic acid (TFSA), in which the *σ* values increased up to ≈3600 S cm^−1^ owing to the highly ordered microstructure with PSS removal. Because the triflate anion from TFSA stably interacted as a counterion to positively charged PEDOT, the PEDOT:PSS–TFSA film presented no significant deterioration of crystallinity during a consecutive reduction process using the tetraenamine‐based reducing agent for *PF* optimization, resulting in the highest *PF* of 534.5 µW m^−1^ K^−2^ with delocalized charge transport properties. Additionally, the high electronic tunability of the reducer enabled the theoretic modeling of the *α–σ* relation over a wide range of *σ*, suggesting that the maximum *PF* value can be higher than that obtained experimentally. To identify the origin of the discrepancy, we analyzed the macro‐ and micro‐scale charge transport properties using the temperature‐dependent *σ*, Hall effects, and magnetoconductance (MC) of the films, together with the morphology, and found that the connectivity between crystalline domains and the resulting high degree of percolation for transport are important factors toward the theoretically ideal *PF*.

## 2 Results and Discussion

### 2.1 Thermoelectric Performance of PEDOT:PSS–TFSA Films

To enhance *σ* of the pristine PEDOT:PSS film, we introduced TFSA (CF_3_SO_3_H) as a post‐treatment agent, which can more effectively protonate PSS (R‐SO3^−^) and remove excess PSSH owing to its strong acidity (pK_a_ = −14.7) in comparison with other acids.^[^ ^34^, ^35^ ^]^ Also, the triflate anion (CF_3_SO_3_^−^, as a conjugate base of TFSA) can form stable electrostatic interaction with positively charged PEDOT, partially resulting in a complex form of PEDOT^+^ and CF_3_SO_3_^−^.^[^ ^35^ ^]^ This is analogous to the molecular structure of PEDOT:trifluoromethanesulfonate (PEDOT:OTf) which is known to have an excellent TE performance and a metallic behavior with high crystallinity.^[^ ^36^, ^37^, ^38^ ^]^ Contrary to the solution‐processible PEDOT:PSS, however, insoluble PEDOT:OTf needs to be synthesized to a solid state via in‐situ polymerization of EDOT monomers using Fe(OTf)_3_, limiting the substrate selectivity, pattern ability, and scalability for practical use. Meanwhile, the PEDOT:PSS film treated with TFSA can be easily formed in a series of solution processes, which can be fabricated in a variety of shapes and sizes, on various substrates, to suit the application. We can also expect the PEDOT:PSS–TFSA film to have excellent thermoelectric and charge transport properties by taking advantage of the triflate anions.

**Figure 1:**
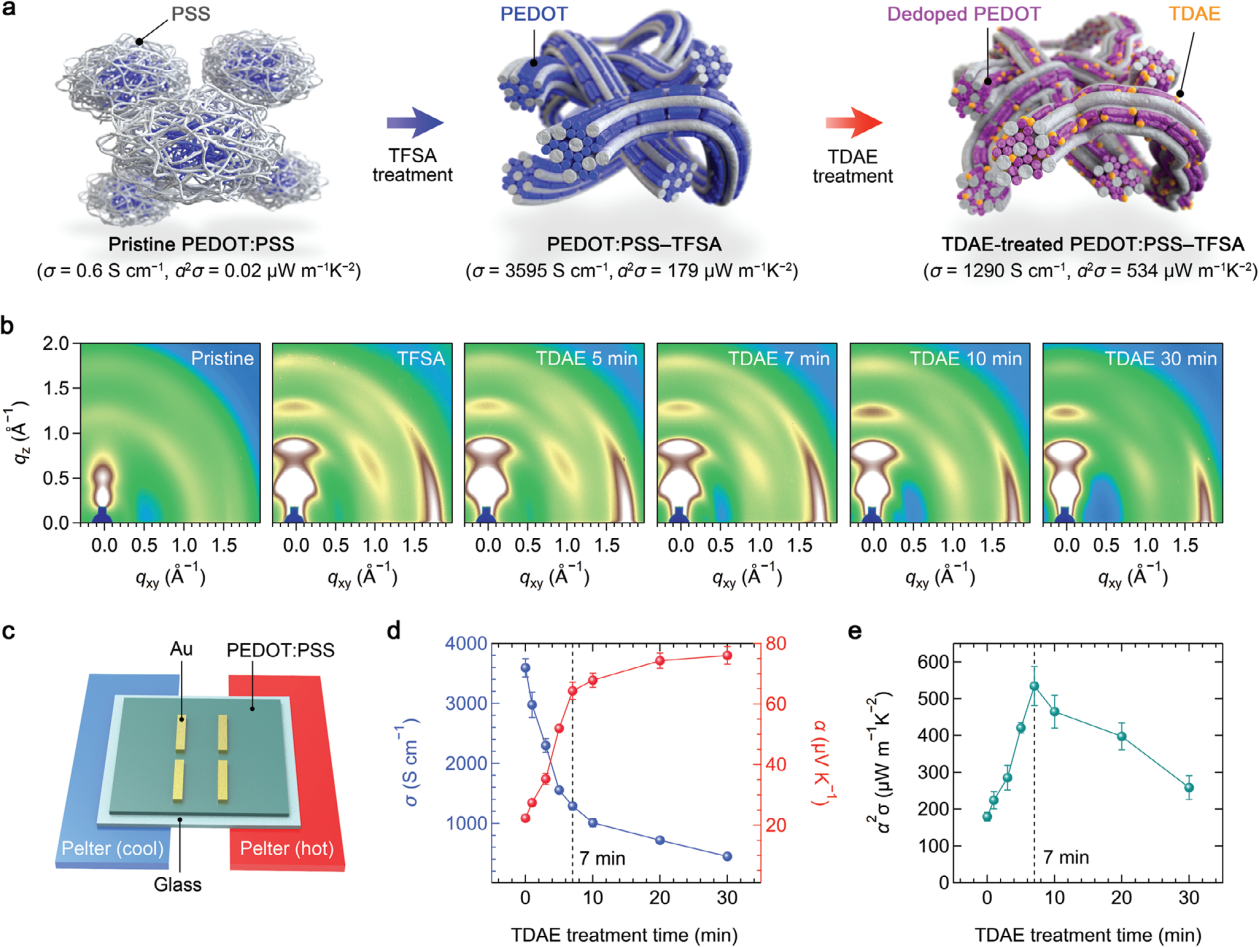
Thermoelectric and structural properties of TDAE‐treated PEDOT:PSS–TFSA films. a) Schematic illustrations of PEDOT and PSS networks in the pristine, PEDOT:PSS–TFSA, and TDAE‐treated PEDOT:PSS–TFSA films. b) 2D GIWAXS patterns of the films along with the TDAE‐treatment time. c) Illustrative structure of the TE devices and measurement. Thermoelectric performance of the PEDOT:PSS–TFSA films in terms of d) *σ*, *α*, and e) *PF* as a function of the TDAE‐treatment time.

The PEDOT:PSS–TFSA films (≈40 nm) were prepared through the TFSA immersion treatment, as described in the Experimental Section. Based on optimization of the treatment conditions (Section S1 and Figure S1, Supporting Information), *σ* was dramatically improved from 0.6 S cm^−1^ in the pristine PEDOT:PSS film to 3595 ± 153 S cm^−1^ in the PEDOT:PSS–TFSA film. Such a high *σ* in a solution‐processed PEDOT:PSS thin film can be achieved not only by the doping effect (i.e., confirmed by the increment of the polaron (≈900 nm) and bipolaron (over 1300 nm) signatures as shown in the UV–vis–NIR absorption spectra in Figure S2 (Supporting Information), but also by the morphological rearrangement (i.e., from the core–shell‐like PEDOT:PSS structure to the well‐aligned fiber‐like PEDOT chains with partial removal of PSS),^[^ ^39^ ^]^ as illustrated in **Figure** **1**a. To verify the formation of the fibrous crystalline PEDOT network in the PEDOT:PSS–TFSA film, we investigated the 2D grazing incident wide‐angle X‐ray scattering (GIWAXS) patterns, as shown in Figure 1b. Contrary to the pristine film, strong consecutive (*l*00) diffraction peaks appeared in the PEDOT:PSS–TFSA films, which is attributed to the alternating lamellar spacing between PEDOT and PSS chains along the out‐of‐plain direction (*q*_z_ = 0.47, 0.91, and 1.34 Å, respectively).^[^ ^40^ ^]^ In addition, a strong (020) diffraction peak can be observed along the in‐plain direction (*q*_xy_ = 1.82 Å), which corresponds to the edge‐on oriented *π*–*π* stacking between PEDOT chains. This alignment of the PEDOT chains is beneficial for achieving high TE performance by facilitating the intra‐ and/or inter‐chain charge transport.^[^ ^41^ ^]^ The TE properties of the films were also measured with a customized measurement setup, as illustrated in Figure 1c, resulting in *α* of 22.3 ± 0.6 µV K^−1^ and the *PF* of 179.5 ± 11.9 µW m^−1^ K^−2^ (Figure S1, Supporting Information) in the PEDOT:PSS–TFSA film with the highest conductivity (3595 S cm^−1^).

To further enhance the *PF* by modulating the doping level of the PEDOT:PSS–TFSA film, we adopted tetrakis(dimethylamino)ethylene (TDAE) as a reducing agent which has a high reducing potential.^[^ ^42^, ^43^ ^]^ Here, we used a TDAE‐vapor treatment method to minimize the deterioration of the film morphology and controlled the oxidation level by varying the treatment time from 0 to 30 min. The de‐doping states could be confirmed by the decrease of the bipolaron state intensity (at >1300 nm) and the work function measured with the UV–vis–NIR absorption spectroscopy (Figure S2a, Supporting Information) and the Kelvin probe force microscopy (KPFM) (Figure S2b,c, Supporting Information), respectively. This was further supported by the X‐ray photoelectron spectroscopy (XPS) spectra. As shown in Figure S2d (Supporting Information), the PEDOT peaks (162–166 eV) consistently shift to lower binding energy as a function of the TDAE treatment time, indicating that the sulfur atoms in PEDOT accepted electrons from TDAE molecules. Notably, the morphology of fibrous PEDOT:PSS networks and the crystallinity of PEDOT chains were well preserved after the de‐doping process, verified with the atomic force microscopy (AFM, see Figure S3, Supporting Information) and the GIWAXS data (Figure 1B; Figure S4, Supporting Information). When the TDAE treatment time was increased, the lamellar stacking distance increased small but consistently from 13.3 Å (*q*_z_ = 0.47 Å^−1^ for 0 min) to 14.5 Å (*q*_z_ = 0.43 Å^−1^ for 30 min), while the *π*–*π* stacking distance remained almost unchanged (≈3.4 Å) (Table S1, Supporting Information). This suggests that the TDAE^+^ molecules infiltrate between PEDOT and counterions, leading to an increase in lamellar spacing while maintaining the *π*–*π* stacking distance of the PEDOT chains (Figure S5, Supporting Information see packing illustration of PEDOT:PSS–TFSA). These arrangements might be attributed to the higher affinity of TDAE^+^ with PSS than with PEDOT.^[^ ^44^ ^]^ In addition, there was no peak splitting or broadening in either the in‐plane or out‐of‐plane directions, indicating that the original lamellar structure and the molecular arrangement in the edge‐on orientation are maintained over the entire de‐doping range. This can be also supported by the fact that the calculated crystal coherence length (CCL), particularly for the *π*–*π* stacking, shows little change after de‐doping (≈1.3 Å, see Table S1, Supporting Information). Therefore, we conclude that the TDAE‐vapor treatment is useful for precisely controlling the electronic state of the PEDOT:PSS–TFSA films without significantly altering the morphological and structural properties. In this way, the PEDOT:PSS–TFSA film with the 7‐min TDAE treatment exhibits a remarkably high *PF* of 534.5 ± 52.8 µW m^−1^ K^−2^ with an increased *α* (64.4 ± 2.8 µV K^−1^) and a decreased *σ* (1289.6 ± 58 S cm^−1^), as plotted in Figure 1d,e.

### 2.2 *α*–*σ* Relationship Using Charge Transport Models

Because the doping level of PEDOT:PSS was tunable in a wide range with the acid–base treatment, we could intensively investigate the *α*–*σ* relationship of the PEDOT:PSS–TFSA films using the Kang–Snyder (K–S) charge transport model.^[^ ^24^ ^]^ Contrary to other transport models,^[^ ^24^, ^25^, ^27^, ^28^ ^]^ this model allows us not only to understand the charge transport behaviors but also to predict a theoretical *PF* maximum from the experimental data of *α* and *σ*.^[^ ^29^ ^]^ For this, we can fit the *σ* and *α* data to the K–S model with the essential fitting parameters constituting the transport function, i.e., the transport coefficient *σ*_E0_ and transport parameter *s* (see Section S2, Supporting Information).^[^ ^24^, ^45^ ^]^ The parameter *σ*_E0_ represents the intrinsic carrier mobility, which is mainly affected by the structural connectivity of the ordered regions in polymers. This value is independent of the doping level of the polymer and reflects the degree of percolation between conductive domains. The transport parameter *s* determines the energy dependence of the transport function, representing the charge transport mechanism of the system. The value of *s* is known to be affected by the microscopic transport properties (i.e., scattering mechanism, local density of states, and relaxation time) of carriers within an individual crystalline polymer domain.^[^ ^46^, ^47^ ^]^

**Figure 2:**
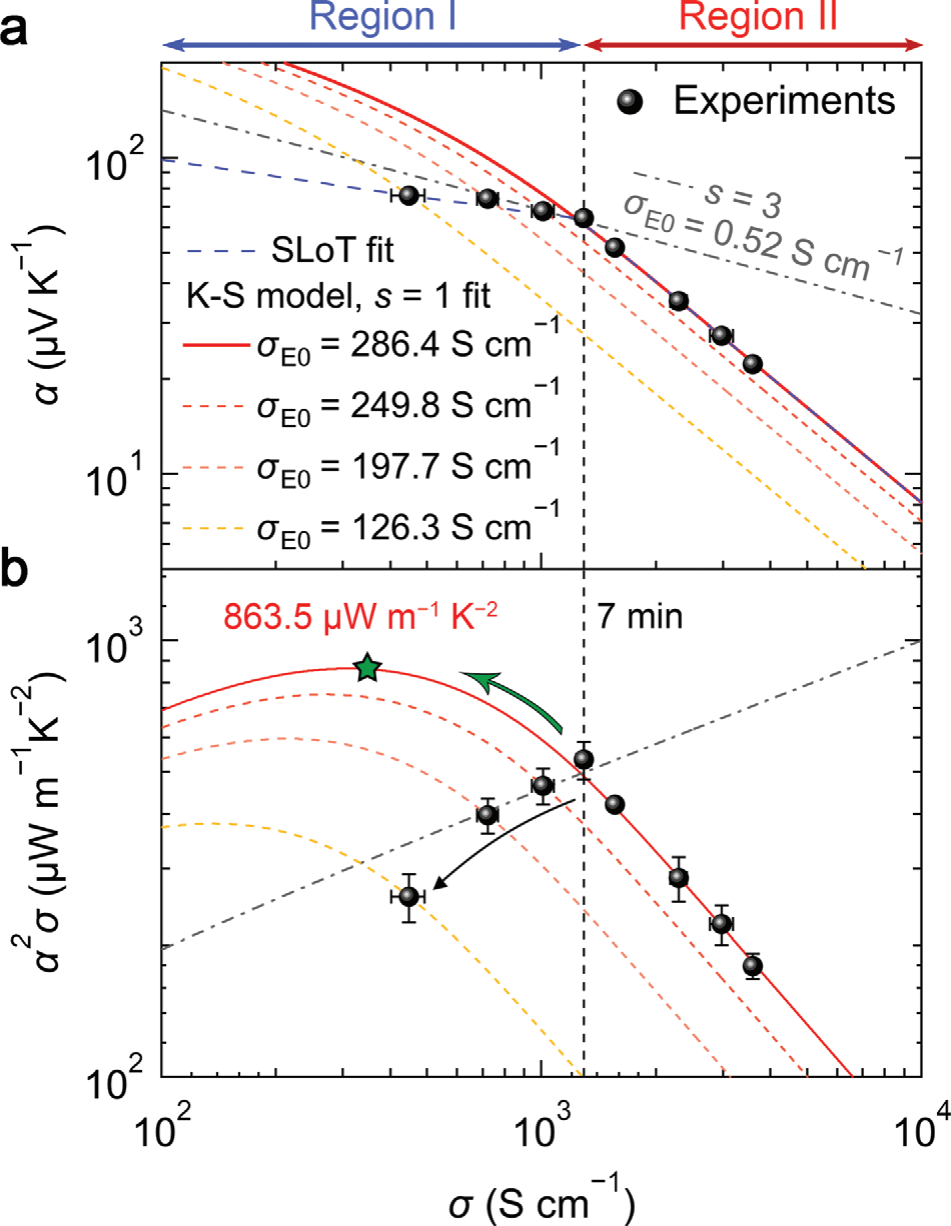
See‐beck coefficient and power factor of the TDAE‐treated PEDOT:PSS–TFSA films as a function of electrical conductivity. a) The *α*–*σ* and b) *α* ^2^ *σ*–*σ* relations of the films are obtained by fitting experimental data to the SLOT and K–S models with various *σ*_E0_ and *s*. The vertical line represents the transition point at which the power law of the *α*–*σ* changes. The power factor begins to decline after seven minutes of de‐doping, failing to achieve the theoretical maximum indicated by the star‐shape marker.

As shown in **Figure** **2**a, the line with *s* = 1 and *σ*_E0_ = 286.4 S cm^−1^ simulated with the K–S model well fits the experimental data in the high conductivity regime (Region II), as divided by a vertical dashed line, specifically when *σ* > 1289.6 S cm^−1^. An excellent fit with *s* = 1 indicates that the transport function is linearly proportional to the energy of the carrier (see Section S2, Supporting Information), which has recently been regarded as the signature for the delocalized charge transport.^[^ ^48^ ^]^ Considering that most doped semiconducting polymers showing thermally activated hopping transport exhibit stronger energy dependency on the transport function (i.e., *s* = 3) with much lower *σ*_E0_ (0.001 to 0.01 S cm^−1^),^[^ ^23^, ^24^, ^49^ ^]^ the high *σ*_E0_ value reaching 286.4 S cm^−1^ reflects the outstanding intrinsic carrier mobility of our PEDOT:PSS system, resulting from the high crystallinity and robust structural connectivity between PEDOT domains as described above in the GIWAXS data. These unique behaviors, which have not been reported to date in simple solution‐processed PEDOT:PSS, maintained well in the film with a TDAE‐treatment time of 7 min, resulting in the highest *PF* of 534.5 µW m^−1^ K^−2^.

On the other hand, the data points at the lower *σ* regime of <1289.6 S cm^−1^ (Region I) deviate from the *s* = 1 relation (Figure 2a). This deviation indicates a transition of the charge transport mechanism, as it cannot be described with a unique transport function (*σ*_E0_ and *s*). To support the change of the transport behavior, we additionally employed the Semi‐localized transport (SLoT) model reported by Yee et al.,^[^ ^25^ ^]^ to fit our data (blue dashed line in Figure 2b) and extracted the value of the localization energy *W*_H_ which is an essential parameter in the transport function of the SLoT model (See the Section S3, Figure S6, and Table S2, Supporting Information). It is notable that the localization energy *W*_H_ was close to zero until the de‐doping time of 7 min, and then abruptly increased after 7 min. The increase in *W*_H_ indicates the increased contribution from hopping, which supports the change in the charge transport behavior. Besides, this transition would have prevented the performance from reaching its theoretical maximum *PF* of 863.5 µW m^−1^ K^−2^, extrapolated from the initial trend of *s* = 1 with *σ*_E0_ = 286.4 S cm^−1^ (see Figure 2b). There are two possibilities accounting for such transition: One is the change of transport mechanism (e.g., metal‐to‐insulator transition or carrier scattering) that can be verified by the change of *s* value from *s* = 1 to *s* = 3 (grey dash–dotted line in Figure 2b), and the other is the decrease in the degree of percolation (e.g., structural connectivity between crystalline domains) that can be verified by a gradual shift of the *s* = 1 curve to the left accompanying a decrease in *σ*_E0_ (dashed lines in Figure 2b). For example, Takenobu et al. showed that the change in *s* represents the metal‐to‐insulator transition using the ion‐gel gated PBTTT films.^[^ ^48^ ^]^ The latter, the decreased percolation between PEDOT domains, is also a reasonable speculation in our case because the de‐doped films still exhibit high *σ* (447.3–1289.6 S cm^−1^) while the *π*–*π* stacking hardly changed from the GIWAXS results. The following sections present systematic examinations of the factors (i.e., transport mechanisms and/or decreases in percolation) that are limited to reaching the theoretical maximum *PF* with the transition of transport. This study includes a thorough analysis of the macroscopic and microscopic properties of charge transport in the films, focusing on the degree of de‐doping.

### 2.3 Transport Mechanisms in PEDOT:PSS–TFSA Films

**Figure 3:**
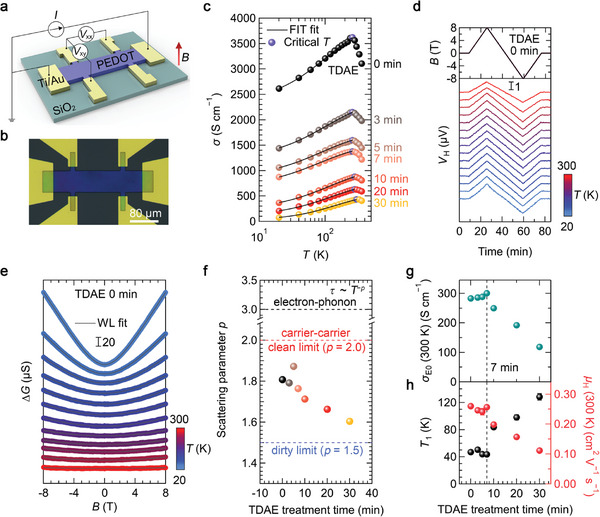
Characterization of charge transport properties of PEDOT:PSS–TFSA films depending on TDAE treatment. a) Schematic illustration of the Hall bar measurement and b) its optical micrograph image of the device. The longitudinal (*V*_xx_) and transverse voltages (*V*_xy_) were recorded simultaneously by applying a constant DC current (*I*). c) The temperature‐dependent *σ* as a function of de‐doping time. The FIT model well fits the experimental data at *T* below the critical temperature. d) The Hall voltage and e) the differential conductance (*∆G* = *G*(*B*) – *G*(0)) when the *B* field was ramped up to 8 T and then down to −8 T at various temperatures from 20 to 300 K. f) Scattering parameter *p* extracted by *τ*–*T* relationship as a function of de‐doping time. g) The transport coefficient *σ*_E0_ of the films when *s* = 1. h) The transport barrier *T*_1_ and Hall mobility *µ*_H_ as a function of the de‐doping time.

We first investigated whether the metal‐to‐insulator transition occurs during de‐doping by measuring the temperature‐dependent electrical conductivity (*σ*(*T*)) and the Hall effect. The PEDOT:PSS–TFSA film fabricated with Hall bar geometry as shown in **Figure** **3**a,b, which allows a simultaneous analysis on *σ*(*T*) and the Hall effect (see Section S4, Supporting Information). We can observe the negative temperature coefficient of *σ* (d*σ*/d*T* <0), which indicates the metallic states,^[^ ^18^, ^48^, ^50^ ^]^ for the PEDOT:PSS–TFSA film above the critical temperature (purple markers), as shown in Figure 3c. Interestingly, this metallic behavior (d*σ*/d*T* <0) is still observable near room temperature even after de‐doping for 30 min. Additionally, our *σ*(*T*) data below the critical temperature exhibit a good fit to the fluctuation‐induced tunneling (FIT) model, *σ*(*T*) = *σ*_0_exp[−*T*_1_/(*T*+*T*_0_)], where *σ*_0_, *T*_1_, and *T*_0_ are the *σ* at the infinite *T*, the transport barrier, and the characteristic temperature, respectively (see Section S4 and Table S3, Supporting Information).^[^ ^41^, ^51^, ^52^ ^]^ This model is commonly used to describe *σ*(*T*) of highly conductive organic materials, such as PEDOT derivatives^[^ ^41^ ^]^ and carbon nanotubes.^[^ ^53^ ^]^ The variable range hopping model^[^ ^54^ ^]^ describing the insulating behavior of polymers fails to fit our *σ*(*T*) data in any dimensions, as confirmed in the Zabrodskii plot (Section S4 and Figure S7, Supporting Information). The delocalized charge transport of the films is further verified by Hall measurement. As shown in Figure 3D, the PEDOT:PSS–TFSA film shows clear Hall voltage signals in a wide range of temperatures from 20 to 300 K, indicating the delocalized nature of charge carriers. Although the sample was de‐doped, a clear Hall voltage still appears, and no sign anomalies were observed (Figure S8, Supporting Information).^[^ ^55^, ^56^ ^]^ These observations including the negative temperature dependence of *σ* as well as the consistent Hall voltage allow us to negate the hypothesis of a metal‐to‐insulator transition.

Subsequently, we expanded the scope of our investigation to examine potential changes in carrier scattering mechanisms—related to the value of *s*—by probing the charge dynamics in a local area via longitudinal magnetoconductance (MC) measurement.^[^ ^24^, ^50^ ^]^ As shown in Figure 3E and Figure S9 (Supporting Information), the PEDOT:PSS–TFSA film and the de‐doped films exhibit a positive MC—the conductance increases in proportion to the magnetic field *B*. The positive MC can be explained by the *B* field‐induced suppression of the weak localization originating from the quantum interference of coherently backscattered electron waves.^[^ ^21^, ^33^, ^57^ ^]^ Importantly, we can reveal the carrier scattering mechanism by analyzing the MC data with the 2D Hikami–Larkin–Nagaoka (H–L–N) model,^[^ ^58^ ^]^

(1) $$\Delta G\left(B\right)\propto -\frac{q^{2}}{2\pi^{2}\hbar }\;\left[\ln\frac{B_{\varphi }}{B}-\Psi \left(\frac{1}{2}+\frac{B_{\varphi }}{B}\right)\right]$$

where *∆G*(*B*) is the conductance difference with and without *B* field, *q* is the unit charge, *ℏ* is the plank constant, *B*_φ_ is the characteristic magnetic field required to destroy phase coherences, and *Ψ* is the digamma function. This model well fits the magnitude and curvature of our MC data (Figure 3e; Figure S9, Supporting Information), which enables us to determine the fitting parameter *B*_φ_. The phase‐breaking time *τ*_φ_ and phase coherence length *λ*_φ_ were calculated using *B*_φ_ = *ℏ*/4*qDτ_φ_*, and *λ_φ_* = (*Dτ_φ_*)^1/2^, respectively, where *D* is the diffusion coefficient defined as *D* = *µ*_H_ *k*_B_ *T*/*q* with the Boltzmann constant *k*_B_. The physical meanings of *τ*_φ_ and *λ*_φ_ are the meantime and distance of travel, respectively, between inelastic collisions while the phase coherence is maintained.^[^ ^33^ ^]^ We can understand the scattering mechanism of the PEDOT:PSS–TFSA films by interpreting the exponent *p*, which is an index determining the *T* dependence of *τ_φ_* as *τ_φ_* ∝ *T* ^−^ *^p^* (Figure S10, Supporting Information).^[^ ^59^ ^]^ For electron–phonon scattering, *p* = 3, and for electron–electron scattering, *p* = 2 (clean limit) and *p* = 1.5 (dirty limit) are expected. As shown in Figure 3F, the samples show only a small decrease of the *p* values from 1.8 to 1.6 along with the de‐doping, but still the values are within the carrier–carrier scattering regime. Our analyses therefore indicate that neither metal‐to‐insulator transition nor changes in carrier scattering mechanism, which are both closely tied to the value of *s*, can account for the observed changes in the transport regime and the limited *PF*. This prompts us to consider the decrease in percolation (expressed as the value of *σ*_E0_) as a possible explanation for the observed transition.

### 2.4 Decreased Percolation in PEDOT:PSS–TFSA Films

We then investigated the effects of the decreased percolation on the transition in the transport regime and the limitation of *PF*, by simultaneously comparing *σ*_E0_, *T*_1_, and the Hall mobility *µ*_H_ upon de‐doping. Figure 3G shows the extracted *σ*_E0_ of each sample when *s* is fixed to 1, indicating that *σ*_E0_ is maintained by the TDAE de‐doping time of 7 min, and then decreases significantly from 300.8 to 118.3 S cm^−1^ when it exceeds 7 min. Since the decrease in *σ*_E0_ implies that the charge transport between PEDOT domains becomes poorer, *T*_1_ and/or *µ*_H_ could be changed significantly. For this, we first obtained *T*_1_ from the FIT model and calculated *µ*_H_ and *n* of the films from the Hall measurement data (Figure S11, Supporting Information). The consistent decrease in *n* (from 8.3 × 10^22^ to 2.4 × 10^22^ cm^−3^ at 300 K) as a function of the TDAE treatment time reaffirms the effective de‐doping of the samples. Figure 3H shows *T*_1_ and *µ*_H_ at room temperature as a function of de‐doping time. The simultaneous comparison of the effects of de‐doping on *T*_1_ and *µ*_H_ enables us to understand how the charge transport between conductive domains changes. The value of *T*_1_ and *µ*_H_ are maintained almost the same until the TDAE de‐doping time of 7 min, and then significantly change after 7 min; the *T*_1_ increases from 43.7 to 128.4 K, and *µ*_H_ decreases from 0.26 to 0.11 cm^2^ V^−1^ s^−1^. Interestingly, the critical de‐doping time for a substantial decline in *σ*_E0_ coincides with the drastic changes in *T*_1_ and *µ*_H_, which may indicate that connectivity between PEDOT domains becomes restricted as the de‐doping occurs. It is also shown that the *PF* limitation occurs at the same de‐doping conditions. The consistent trends observed across various parameters, including *σ*_E0_, *T*_1_, and *µ*_H_, provide compelling evidence that decreased percolation plays a pivotal role in the transition in transport regime and *PF*. In the subsequent section, we aim to elucidate the reasons for the reduction in percolation due to de‐doping, which can offer several strategies for reaching the theoretical maximum of *PF*.

### 2.5 Origin of Transition in Charge Transport and PF

**Figure 4:**
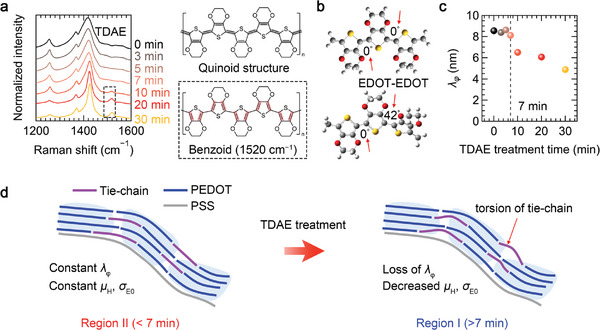
Origin of transition in charge transport regime and power factor a) Normalized Raman spectra as a function of TDAE treatment time. The dotted rectangular indicates the appearance of the benzoid structure. b) Structural optimization of the three EDOT units in a single PEDOT chain in the quinoid and benzoid states. The numbers are dihedral angles between the adjacent of EDOT. c) Inelastic coherence length *λ*_φ_ as a function of de‐doping time. d) Schematic illustration depicting the change in structural connectivity by tie‐chain torsion in PEDOT:PSS after 7‐min duration of de‐doping. The tie‐chain torsion degrades the charge transport between crystalline domains.

It is well understood that the macroscopic charge transport in semi‐crystalline polymers is mediated by tie‐molecules, which act as intermediaries between crystalline domains.^[^ ^48^, ^60^, ^61^ ^]^ The decrease in percolation, therefore, could be attributed to structural alterations in these tie‐molecules induced by de‐doping after 7 min. Although interchain coupling within PEDOT domains may also be able to hinder percolation for transport,^[^ ^41^, ^61^ ^]^ this speculation is not relevant to our study, as confirmed by the constant *π*–*π* stacking distance of PEDOT chains via GIWAXS data. Also, the increased lamellar distance seems to have a limited effect on the reduced percolation, because the carriers are mainly transported through the *π*–*π* chains,^[^ ^60^, ^62^, ^63^ ^]^ and the *d*‐spacing value behaves differently upon doping compared to the transport parameters, as shown in Table S1 (Supporting Information). To observe the structural change in the PEDOT molecules, we examined the Raman spectra of the films as shown in **Figure** **4**a. Notably, a peak at 1530 cm^−1^ emerges and intensifies after 7 min of de‐doping (marked by the dotted rectangular), indicating the transformation of PEDOT chains from a planar, rigid quinoid structure to a less planar, flexible benzoid structure.^[^ ^64^, ^65^ ^]^ We then performed density functional theory (DFT) calculations to investigate an optimal configuration of an isolated PEDOT molecule consisting of the three EDOT units (Figure 4b). The calculations revealed that the torsion between the EDOT–EDOT bond in the benzoid configuration is higher than that in the quinoid one. When this conformational change occurs in tie‐molecules upon de‐doping, it deteriorates their on‐chain charge transport ability, thereby being able to hinder the interdomain transport even in highly ordered polymer systems. The effects of the conformational change on interdomain transport can be elucidated by examining the change in phase coherence length *λ*_φ_, an important parameter that enables to detection of the local transport properties.^[^ ^50^, ^57^ ^]^ As shown in Figure 4c, the *λ_φ_* value in our PEDOT:PSS–TFSA films remain almost constant (≈8 nm) until the TDAE de‐doping time of 7 min, but it abruptly decreases after that, reaching down to 5 nm at the de‐doping time of 30 min. Assuming the length of individual PEDOT chain being roughly 3–7 nm,^[^ ^66^ ^]^ the abrupt decrease in *λ*_φ_ to this value suggests that the charges are localized within each PEDOT domain. Such loss in the phase coherence serves as evidence that the macroscopic charge transport is limited at the domain boundaries. It also provides a justification for the aforementioned change in the macroscopic parameters including *σ*_E0_, *T*_1_, and *µ*_H_. Overall, through an in‐depth examination of the macro‐ and micro‐scale charge transport, we found that the conformational changes in tie‐chain of PEDOT molecules by de‐doping mainly limits the percolation for long‐range transport, hindering the achievement of the theoretical maximum *PF* (Figure 4d).

## 3 Conclusion

This work demonstrates solution‐processable, high‐performance TE devices based on optimally acid–base‐treated PEDOT:PSS thin films. Our sequential treatment by using super‐acid and base has enabled the films to have a highly ordered microstructure even after the de‐doping process, leading to efficient long‐range delocalized charge transport with a high *PF*. The transport analysis using an energy‐dependent transport function model allows us to predict the ideal *PF* maximum, guiding us toward further optimizing the *PF* value. Through a comprehensive examination of the structure–property relationships at both macro‐ and micro‐scales, we reveal that conformational changes particularly in tie‐molecules induced by prolonged base treatment ultimately hinder long‐range charge transport between inter‐PEDOT domains, leading to *PF* limitation. Our approaches not only offer insights into the structure–property relationship of solution‐processable PEDOT:PSS, but also pave the way for the development of high‐performance TE materials for the future advancement of energy harvesting technologies.

From the results, we highlight two central insights: First, higher crystallinity and more robust structural connectivity of conjugated polymer film are critical to achieving higher *PF* values through enhanced long‐range delocalized charge transport. Second, the minimization of conformational disorders provides an effective strategy to reach the theoretical maximum *PF*, particularly by mitigating the decrease in *λ*_φ_. For this, we propose several strategies, such as removing remaining PSS and/or residual dopants, enhancing intrachain rigidity and crystallinity in PEDOT:PSS films, and exploring de‐doping by novel n‐type dopants with high doping efficiency—capable of significantly adjusting the doping level of a polymer with even minimal quantities, to prevent the residue of dopant molecules that interfere with charge transfer. (Figure S12, Supporting Information). Importantly, the methodologies and principles outlined in this study can be extended to other heavily doped polymers, thereby opening up new avenues for the development of high‐performance TE materials with elevated *PF* values.

## 4 Experimental Section

### Sample Preparation

PEDOT:PSS aqueous solution (Clevios PH 1000, Heraeus), TFSA (TCI chemicals), methanol, and TDAE (Sigma–Aldrich) were purchased and used as received. For the film preparation, all substrates (i.e., bare glass, ITO‐coated glass, quartz, and Si/SiO_2_ wafer) were ultrasonically cleaned in deionized water, acetone, and isopropyl alcohol, followed by drying in a vacuum oven for 12 h. After UV–ozone surface treatment, a PEDOT:PSS film was deposited by spin‐coating at 2300 rpm for 20 s, and then annealed at 100 °C for 10 min. The second PEDOT:PSS layer was sequentially formed under the same conditions, resulting in a total thickness of 50 nm. For the acid treatment, the pristine PEDOT:PSS film was immersed in a TFSA bath for 1 min. After that, the film was immediately rinsed with methanol to remove residual TFSA, followed by annealing at 150 °C for 5 min. This procedure was repeated to optimize electrical properties (Figure S1, Supporting Information). For the base treatment, the as‐prepared sample was placed in a pre‐heated (100 °C), home‐built vacuum chamber filled with TDAE vapor. The reduction level was controlled by changing the treatment time. To measure the TE properties, Au electrodes (70 nm) were deposited on the films by thermal evaporation under a high vacuum (≈3 × 10^−7^ torr) with a deposition rate of 1.0 Å s^−1^ through a patterned shadow mask. Two electrodes (17 mm × 8 mm) for the hot and cold sides were separated by 2 mm. For the Hall effect and magnetoconductance measurements, the electrodes composed of Ti (7 nm) and Au (70 nm) were e‐beam evaporated onto the SiO_2_ substrate and lithographically patterned by the conventional lift‐off process. After UV–ozone treatment, PEDOT:PSS was deposited and treated with acid and/or base, in the same way described above. Parylene‐C (1 µm) was then deposited onto the films using a parylene deposition system (PDS 2010, SCS Inc.) to protect the active layer from the subsequent photolithography process to pattern the active layer into the precise hall bar geometry by oxygen plasma etching (150 W for 5 min).

### Characterization

UV–vis absorption was measured using a JASCO V‐770 spectrophotometer. GIWAXS data were obtained at the 9A U‐SAXS beamline at Pohang Accelerator Laboratory (Pohang, Republic of Korea). Thickness and surface morphology of the films were characterized using a non‐contact mode AFM (Veeco, Nanoscope IV). The local currents of the films were measured with the contact mode C‐AFM using a PT/Ir‐coated cantilever (SCM‐PIT‐V2). The work functions of the films were characterized by analyzing the surface potential contrast (SPC) data obtained from KPFM using the same cantilever. XPS measurements were conducted using an AXIS HSi spectrometer employing a monochromatic Al Kα light source. The *σ*(*T*), Hall effect, and magneto‐conductance were measured using a physical property measurement system (PPMS, model PPMS‐14, Quantum Design, USA) installed at the National Center for Inter‐university Research Facilities (NCIRF) at Seoul National University. The longitudinal and transverse voltages were recorded simultaneously while the magnetic field was swept from 8 T to −8 T at the rate of 0.5 T min^−1^. The electrical conductivity *σ* was calculated with the equation *σ* = 1/(*R*_s_×*t*), where *R*_s_ is the surface resistance measured by the four‐point probe method and *t* is the thickness of the film. The Seebeck coefficient was measured from the home‐made stage composed of two Peltier modules controlled with the Keithley 2604B source meter. Two T‐type thermocouples connected to the Keithley 2700 multimeter were attached to the hot and cold sides of the films to detect the temperature. The generated thermovoltage was measured using the Keithley 2182A nanovoltmeter. The Seebeck coefficient was extracted by the linear‐fitting the pairs of the measured Δ*T* and Δ*V*. Density functional theory calculations were executed using the Gaussian 09 program with a B3LYP functional and /6‐31G(d,p) basis set.

## Conflict of Interest

The authors declare no conflict of interest.

## Supporting information

Supporting Information

## Data Availability

The data that support the findings of this study are available from the corresponding author upon reasonable request.
