# Supplementary material for: High Thermoelectric Performance in Solution‐Processed Semicrystalline PEDOT:PSS Films by Strong Acid–Base Treatment: Limitations and Potential
Source: Adv Sci (Weinh). 2024 Jan 18;11(10):2308368. doi: 10.1002/advs.202308368 (PMC10933597; doi:10.1002/advs.202308368)
Supplement: Supplementary file 1 — Supporting Information [file ADVS-11-2308368-s001.pdf]

ADVANCED SCIENCE
Open Access

Supporting Information

for *Adv. Sci.*, DOI 10.1002/advs.202308368

High Thermoelectric Performance in Solution-Processed Semicrystalline PEDOT:PSS Films by Strong Acid–Base Treatment: Limitations and Potential

Juhyung Park, Jae Gyu Jang, Keehoon Kang, Sung Hyun Kim and Jeonghun Kwak**

Supporting Information

High Thermoelectric Performance in Solution-Processed Semicrystalline PEDOT:PSS Films by Strong Acid–Base Treatment: Limitations and Potential

Juhyung Park†*, Jae Gyu Jang*†*, Keehoon Kang, Sung Hyun Kim*, Jeonghun Kwak**

†These authors equally contribute to this work
*Corresponding authors.

J. Park, Prof. J. Kwak
Department of Electrical and Computer Engineering, Inter-university Semiconductor Research Center, Seoul National University, Seoul 08826, Republic of Korea
E-mail: jkwak@snu.ac.kr

J. G. Jang, Prof. S. H. Kim
Department of Carbon Convergence Engineering, Wonkwang University, Iksan 54538, Republic of Korea.
E-mail: shkim75@wku.ac.kr

Prof. K. Kang
Department of Materials Science and Engineering, Research Institute of Advanced Materials, and Institute of Applied Physics, Seoul National University, Seoul, 08826, Republic of Korea.

Section S1. Optimization of TFSA acid treatment

To improve σ of the PEDOT:PSS films, we measured the sheet resistance of the TFSA-treated PEDOT:PSS films with varying the number of TFSA treatments (Figure S1). The sheet resistance of the films was slightly decreased from 58.1 ± 1.8 ohm sq^{-1} to 50.8 ± 2.2 ohm sq^{-1} as the number of treatments increased from one to three times. As the number of treatments further increased to five times, the R_s was increased to 91.5 ± 0.3 ohm sq^{-1}. Meanwhile, the thickness of the films gradually decreased from 64.6 ± 4.4 nm to 39.2 ± 2.4 nm when the films were treated from one to five times. Consequently, the film exhibited a maximum σ of 3595.1 ± 153.4 S cm^{-1} after three times of TFSA treatments. The Seebeck coefficient of the films slightly decreased from 23.9 ± 2.2 μV K^{-1} to 20.6 ± 1.3 μV K^{-1} by increasing the number of treatments from one to five times. The highest power factor was obtained when the film was treated for three times, showing 179.5 ± 11.9 μW m^{-1} K^{-2}.

Section S2. Kang–Snyder model

Kang–Snyder modelling. σ and α can be generally characterized by the transport function $\sigma_E(E)$ describing the capability for electrical conduction at each energy level in the units of σ.[1, 2] By solving the Boltzmann transport equations, the α and σ are expressed with $\sigma_E(E)$ as the following equations:

$$\sigma = \int \sigma_E \left(-\frac{\partial f}{\partial E}\right) dE \tag{S1}$$

$$\alpha = \frac{k_B}{q} \int \frac{(E - E_F)}{k_B T} \frac{\sigma_E}{\sigma} \left(-\frac{\partial f}{\partial E}\right) dE \tag{S2}$$

Here, f is the Fermi–Dirac distribution function, $f = 1/[1+\exp\{(E-E_F)/(k_BT)\}]$, where q, k_B and E_F are a unit charge, Boltzmann constant, and chemical potential of the polymer, respectively. The Kang–Snyder model suggests a novel form of transport function,[1] which has power law energy dependency with energy E above the transport edge E_t,

$$\sigma_E(E,T) = \begin{cases} 0 \ (E < E_t) \\ \sigma_{E0} \times \left(\frac{E - E_t}{k_B T}\right)^s \ (E \geq E_t) \end{cases} \tag{S3}$$

where $\sigma_{E0}(T)$ and s are the transport coefficient and transport parameter, respectively. By replacing the transport function $\sigma_E(E)$ (Equation S1 and S2) with Equation S3, the simplified form of σ and α can be obtained:

$$\sigma = \sigma_{E0}(T) \times sF_{s-1}(\eta) \tag{S4}$$

$$\alpha = \frac{k_B}{q}\left[\frac{(s+1)F_s(\eta)}{sF_{s-1}(\eta)} - \eta\right] \tag{S5}$$

Here, $F_i(\eta)$ is the non-normalized complete Fermi-Dirac integral, and $\eta = (E_F - E_t)/k_BT$ is the reduced chemical potential. The above equations (Equation S4, S5) enable us to determine σ_{E0}, s and η from experimentally obtained σ and α.

Section S3. Semi-localized model

Recently, Yee et al. presented the semi-localized transport model by modifying the transport function of the K–S model.[3] The SLoT model well captures the transition in charge transport behavior in conjugated polymers as a function of carrier concentration ratio c. The transport function of SLoT model is expressed as:

$$\sigma_E(E,T,c) = \begin{cases} 0 \ (E < E_t) \\ \sigma_0 \exp\left(-\frac{W_H(c)}{k_B T}\right) \times \left(\frac{E - E_t}{k_B T}\right) \ (E \geq E_t) \end{cases} \quad (S6)$$

In this equation, W_H represents the localization energy, which correlates with both spatial and electrostatic effects. This function differs from the K–S model by introducing an additional term $\sigma_0\exp(-W_H(c)/(k_BT))$, representing an Arrhenius-like hopping contribution from the charge carriers. The SLoT model also sets the transport parameter s to 1. As the carrier concentration ratio c exceeds c_d (the carrier ratio to achieve delocalized transport), W_H converges to zero, making the transport function of the SLoT model equivalent to the $s = 1$ case in the K–S model. Conversely, when c falls below c_d, W_H increases, causing the transport function to deviate from the $s = 1$ fit observed in the K–S model.

To fit our data using SLoT model, we employed the "SLoTModel.xlsx" file provided by the authors.[3] The modelling parameters (e.g., c_{max}, A_0, A_1, $W_{H,max}$, $W_{H,slope}$) and material parameters (e.g., M_0, N, ρ, k_BT) are detailed in Table S2. Here, c_{max} is the maximum c value needed for the data set, A_0 and A_1 are fitting parameters, $W_{H,max}$ is the maximum localization energy, and $W_{H,slope}$ represents the rate at which localization diminishes as c increases. M_0 denotes the molecular weight of the monomer unit, and N is the number of sites per monomer ratio, and ρ is the density of the polymer.

Section S4. Experimental details for measuring temperature-dependent electrical conductivity and magnetotransport properties

Device fabrication. Temperature-dependent electrical conductivity and magnetotransport properties were measured using Hall bar-structured devices, where PEDOT:PSS layers were precisely patterned to enable accurate measurement of local potentials. The fabrication process for the Hall bars included several key steps. Initially, a Si/SiO_2 substrate was cleaned through a sonication processes in deionized water, followed by additional rinsing in acetone and isopropanol. Electrodes consisting of titanium (Ti, 7 nm) and gold (Au, 70 nm) were deposited onto the Si/SiO_2 substrate through e-gun evaporation, followed by patterning using standard lift-off lithography techniques. The dimensions for the channel length (L) and width (W) were 240 μm and 60 μm, respectively. Four probes were positioned between source and drain electrodes, with the distance between two longitudinal probes along the channel length (defined as L^*) designed to be 110 μm. The width of the probe was 15 μm. After electrodes formation, the PEDOT:PSS layer was spin-coated onto the Si/SiO_2 substrate, and sequential acid–base treatments were applied as described in the manuscript. Subsequently, a parylene-C layer (1 μm) was deposited onto the PEDOT:PSS films using a parylene coater (PDS2010), to protect the active layer during subsequent processing. The active layer was then patterned into precise Hall bar geometry through photolithography and oxygen plasma etching at 150 W for 5 min. Both photoresist and parylene-C were remained as a protective layer for the active channel. All fabrication steps were conducted in a cleanroom environment with controlled humidity and temperature. For the magnetotransport measurements using a PPMS system, a ball bonder was utilized for wiring the hall bar device to the PPMS puck.

Temperature-dependent electrical conductivity of the PEDOT:PSS–TFSA films. The temperature-dependent electrical conductivity $\sigma(T)$ was evaluated from the same hall bar geometry using the formula $\sigma = IL^*/(\Delta V_{xx}Wt)$, where t represents the thickness of the active layer. For all PEDOT:PSS films, a negative T dependence of σ ($d\sigma/dT < 0$) was observed, indicating the metallic behaviors above the critical temperature. Below this critical temperature, the thermally activated type of σ ($d\sigma/dT > 0$) is observed. The $\sigma(T)$ of our samples at low temperature aligns well with the fluctuation-induced tunneling (FIT) model: $\sigma = \sigma_0\exp\{-T_1/(T + T_0)\}$, where σ_0 is the σ at infinite T, and T_0 is the characteristic temperature, respectively.[4,5] In this model, thermally activated voltage fluctuations across insulating

regions are critical for electrical conduction. Parameters derived using the FIT model are summarized in Table S3.

We also investigated the $\sigma(T)$ behavior of our PEDOT:PSS films using the variable range hopping (VRH) model, $\sigma = \sigma_0 \exp(T_a/T)^{-1/m}$, which is generally adopted to explain the thermally-activated type of $\sigma(T)$ of the conjugated polymers,[6] where σ_0 is the σ at the infinite T, and T_a is the characteristic temperature. Here, m is the crucial factor that determines the charge transport dimensionality. The m-values were determined from Zabrodskii plots ($W = \Delta\ln\sigma/\Delta\ln T$ vs. T) where the slope is equal to the value of $-1/m$, as illustrated in Figure S7. Interestingly, we found that none of the m-values from our samples conformed to the standard VRH dimensions of m = 2, 3, or 4. This observation suggests that the thermally activated behaviors in our PEDOT:PSS films cannot be explained by the localized state hopping mechanisms.

Calculation of the electrical parameters extracted by the Hall effect measurements. The Hall coefficient R_H, Hall mobility μ_H, and Hall carrier concentration n_H were obtained using the following equations:

$$R_{\mathrm{H}} = \frac{dV_{\mathrm{H}}}{dB} \times \frac{t}{I} \quad (S7)$$

$$\mu_{\mathrm{H}} = |R_{\mathrm{H}}|\sigma \quad (S8)$$

$$n_{\mathrm{H}} = \frac{1}{eR_{\mathrm{H}}}, \quad (S9)$$

The actual Hall voltage V_H was corrected by subtracting both the field-independent and field-dependent offset voltages arising from the positive magnetoconductance from the measured ΔV_{xy}, as reported previously.[7] The extracted V_H for TDAE-treated films is presented in Figure S8, while the temperature-dependent σ, μ_H, and n_H are also shown in the Figure S11.

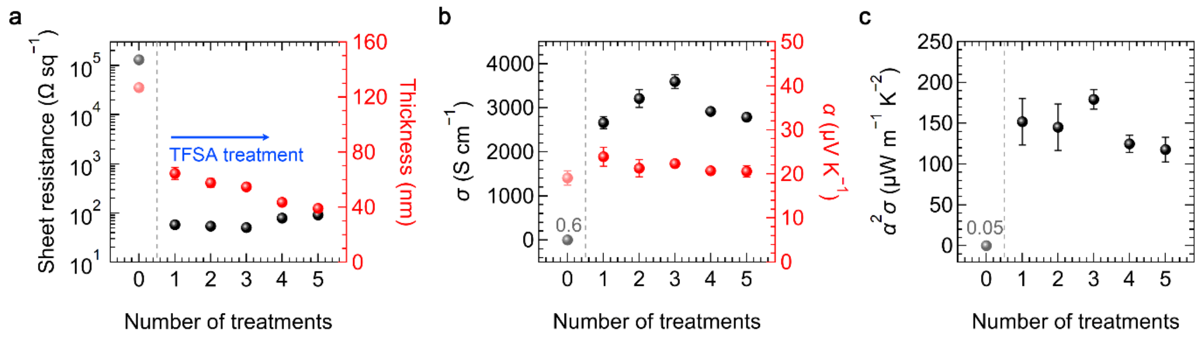

Figure S1. Optimization of TE performance through super-acid treatments. a) Sheet resistance and thickness, b) σ and α, and c) $\alpha^2\sigma$ of the PEDOT:PSS–TFSA films according to the number of acid treatments.

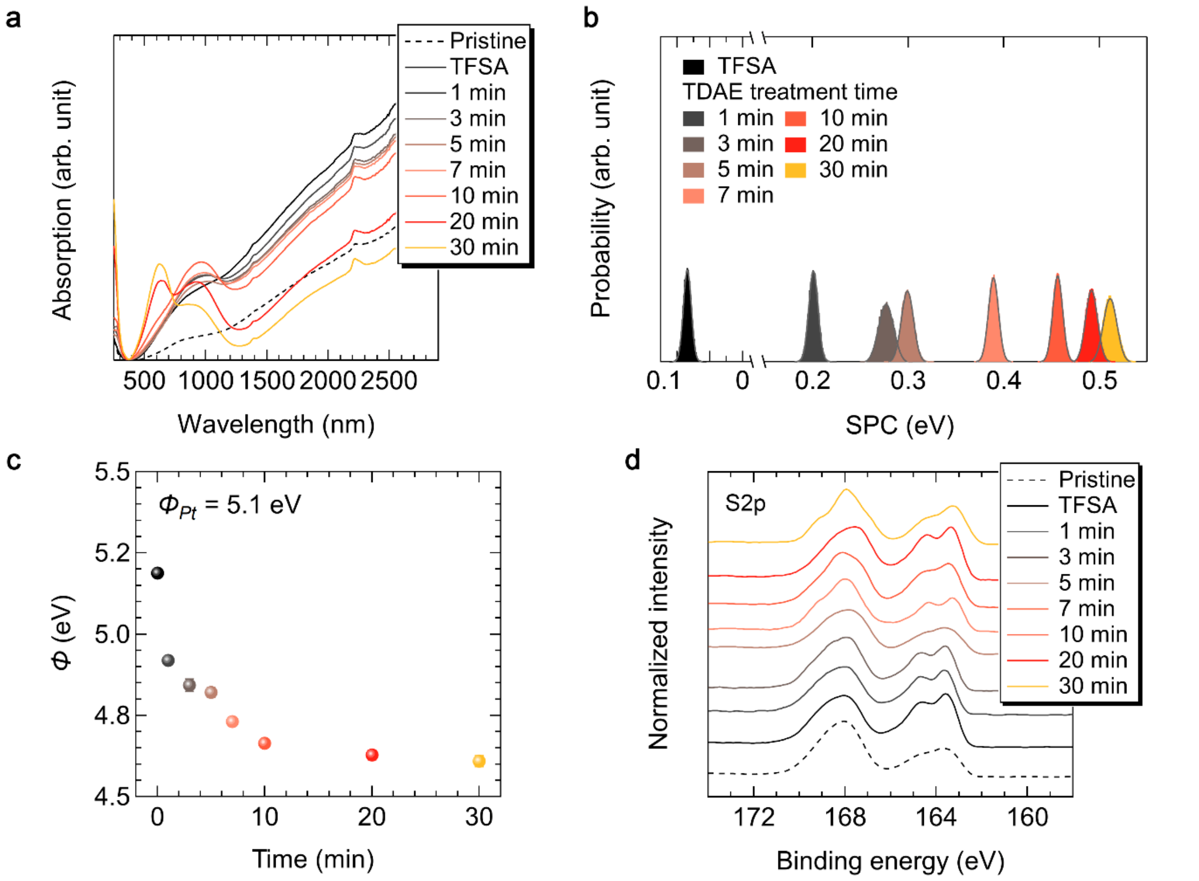

Figure S2. Effects of the acid–base treatments on the electronic structure of the films. a) UV–Vis–NIR absorption spectra of the pristine PEDOT:PSS film and the PEDOT:PSS–TFSA films subjected to TDAE treatment time. Spectral intensities around 620 nm, 900 nm, and 1300 nm correspond to the neutral, polaron, and bipolaron states of the PEDOT chains, respectively. b) SPC histograms and c) work functions for the PEDOT:PSS–TFSA films with different TDAE treatment time. The work functions determined by fitting the SPC spectra using a Gaussian function. d) S2p XPS spectra of the pristine PEDOT:PSS film and the PEDOT:PSS–TFSA films subjected to TDAE treatment time. Two S2p peaks are shown in the regions of 162–166 eV and 167–172 eV, corresponding to thiophene units in PEDOT and sulfonic acid units in PSS, respectively.[8] Following the TFSA treatment, the relative peak intensity of PEDOT to PSS increased noticeably, indicating the removal of PSS.

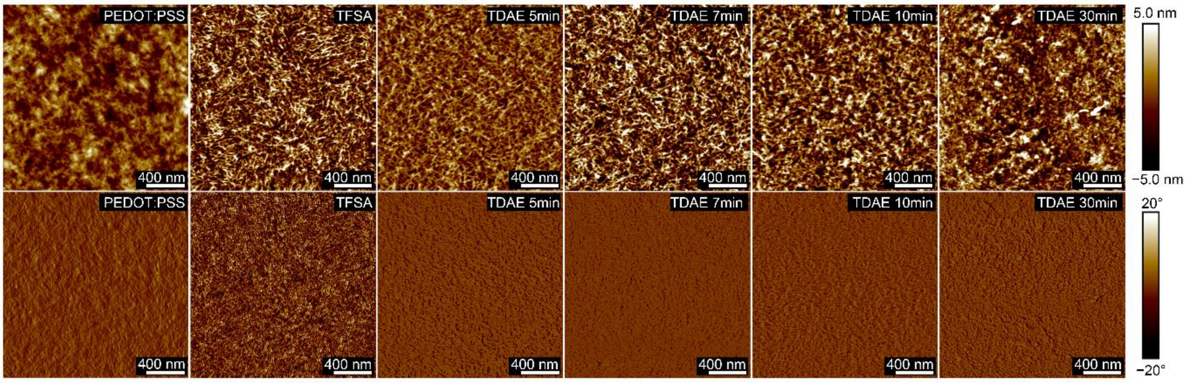

Figure S3. Morphology change of the PEDOT:PSS films. AFM topography and phase images of the PEDOT:PSS films measured in a size of 2 μm × 2 μm.

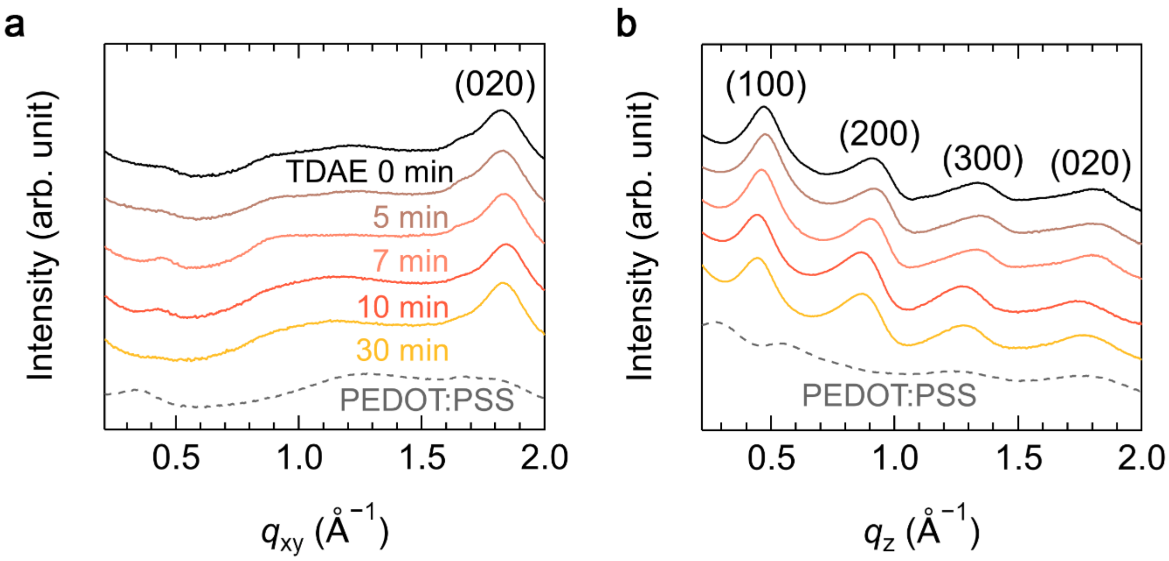

Figure S4. Line-cut profiles obtained from 2D GIWAXS patterns. a) In the q_{xy} direction and b) in the q_z direction, for both pristine PEDOT:PSS film and PEDOT:PSS–TFSA films as function of TDAE treatment time. The pristine PEDOT:PSS film exhibits characteristic peaks at $q \approx 0.27$ Å^{-1}, 0.51 Å^{-1}, 1.19 Å^{-1}, and 1.80 Å^{-1} in the q_z direction. These peaks correspond to the lamellar stacking of PEDOT and PSS, the amorphous halo of PSS, and the π–π stacking of PEDOT (i.e., the (020) planes of PEDOT), respectively.

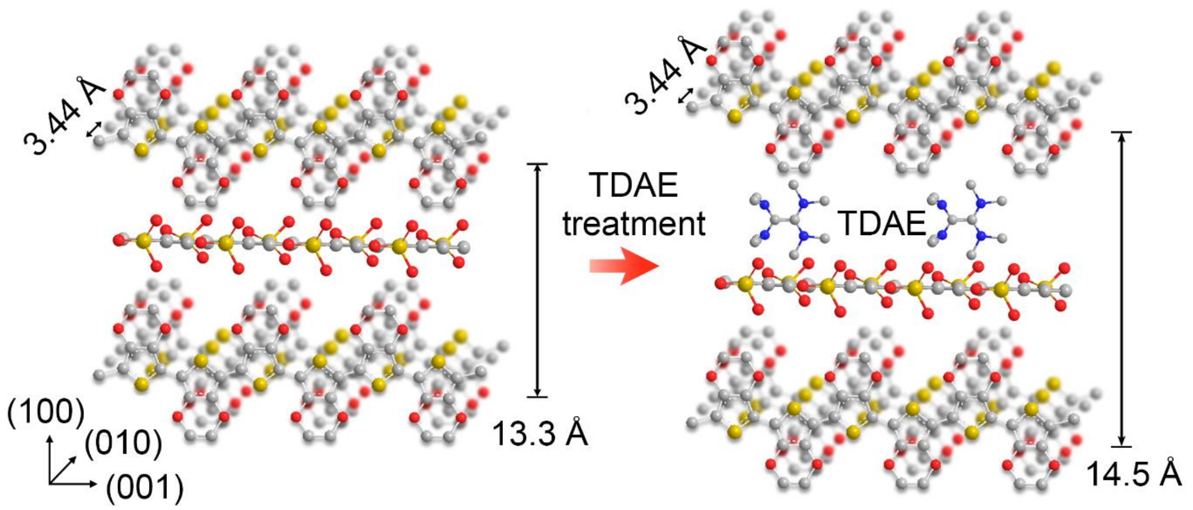

Figure S5. Schematic illustration of packing arrangement changes in PEDOT:PSS–TFSA films after TDAE treatment. The lamellar stacking distance between PEDOT and PSS expands from 13.3 Å to 14.5 Å after the TDAE treatment (30 min), while the *π*–*π* stacking of PEDOT remains constant at 3.44 Å.

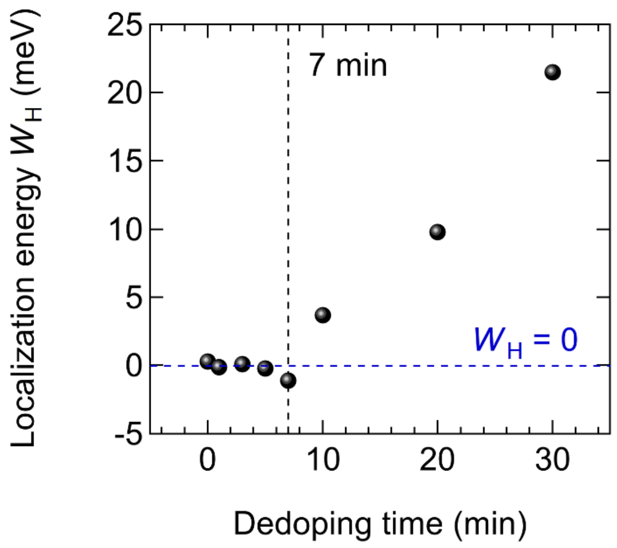

Figure S6. Change in localization energy W_H as a function of TDAE treatment time. The values of W_H for the samples were extracted by the SLoT model. When the W_H converges to zero, the transport function of SLoT model becomes equivalent to the $s = 1$ case in the K–S model. After the de-doping time exceeds the 7 min, the W_H undergoes a sudden increase, supporting the deviation of the $s = 1$ fit of our experimental data.

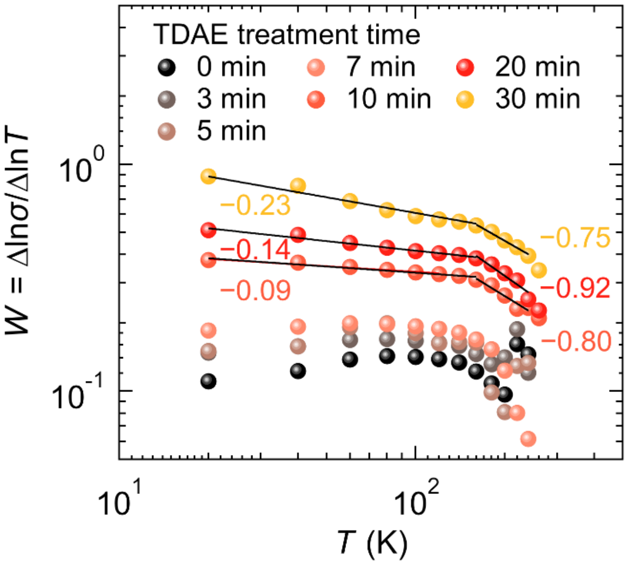

Figure S7. Zabrodskii plot of the TDAE-treated PEDOT:PSS–TFSA films. The slope of the fit line equals to the value of $-1/m$, which gives the information of the dimension m of the VRH mechanism.

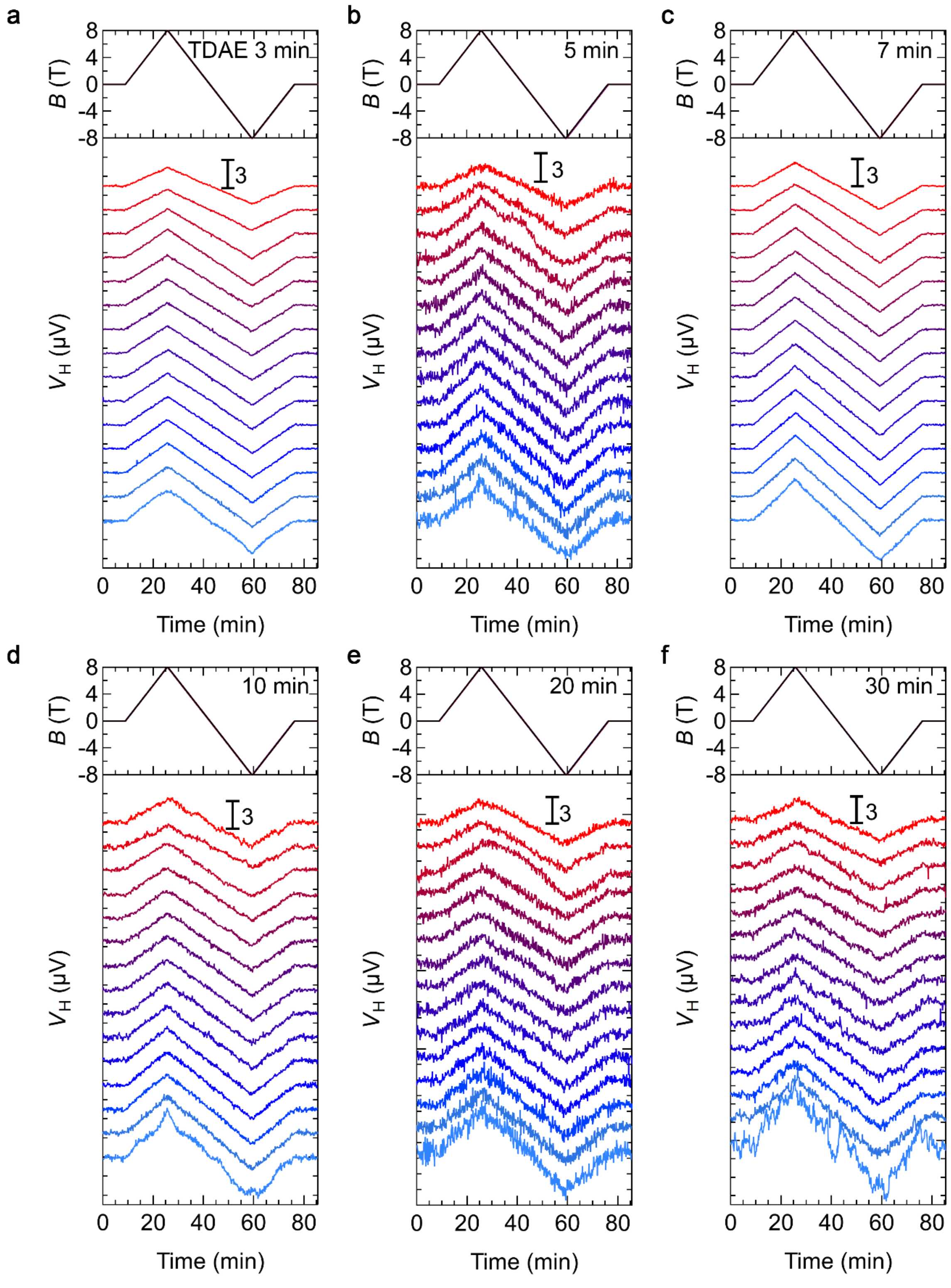

Figure S8. Effects of *B*-field on the Hall voltage of the TDAE-treated PEDOT:PSS–TFSA films. a–f) The extracted Hall voltages of the PEDOT:PSS–TFSA films subjected to de-doping times ranging from 3 to 30 min. The *B* field was ramped up to 8 T and down to −8 T at various temperatures from 20 K to 300 K.

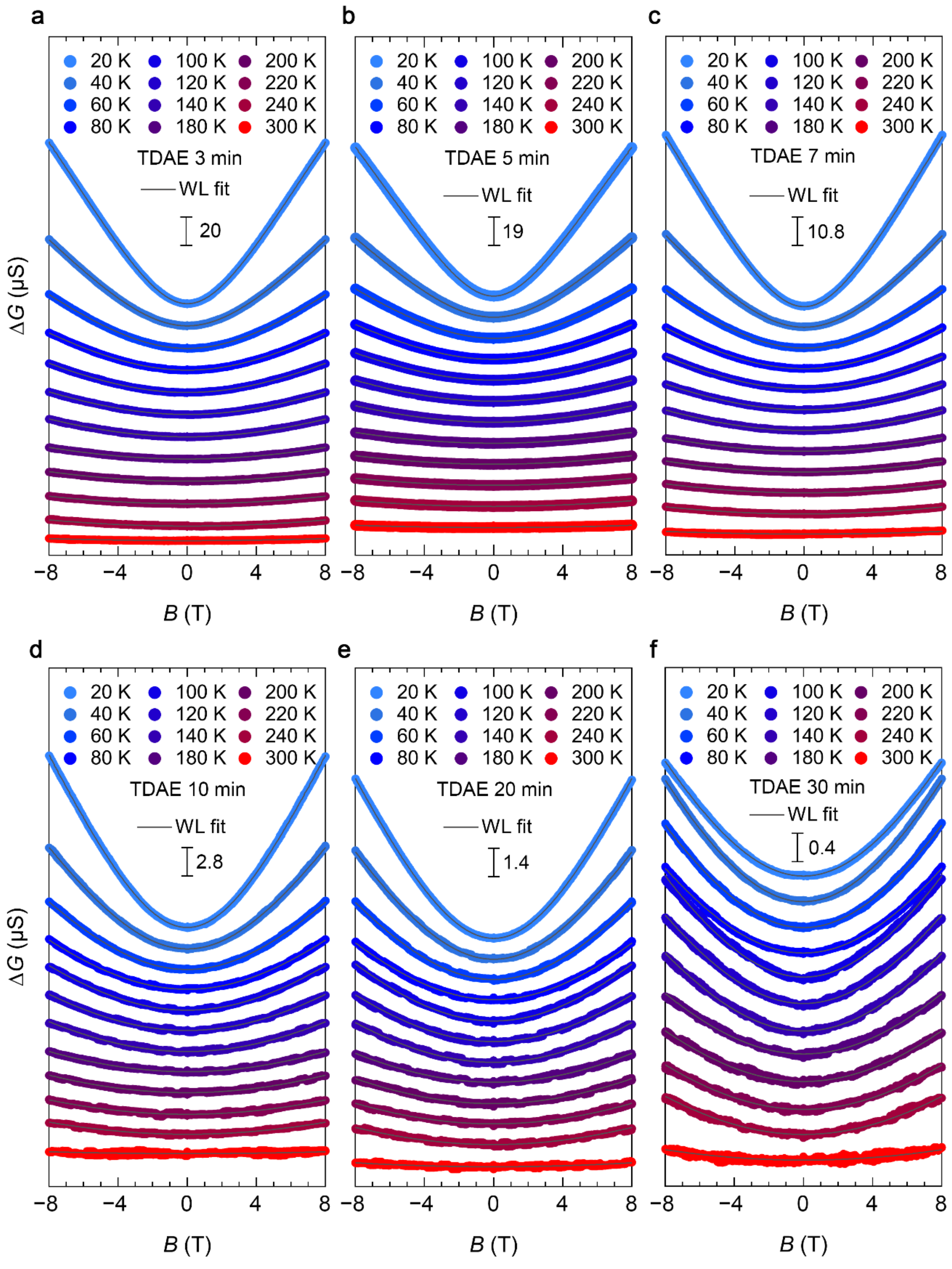

Figure S9. Weak localization in the magnetoconductance of the TDAE-treated PEDOT:PSS–TFSA films. Effect of *B* field applied perpendicular to the substrate on differential conductance ($\Delta G = G(B) - G(0)$) of the films at a various temperature; a) 3 min, b) 5 min, c) 7 min, d) 10 min, e) 20 min, and f) 30 min, respectively.

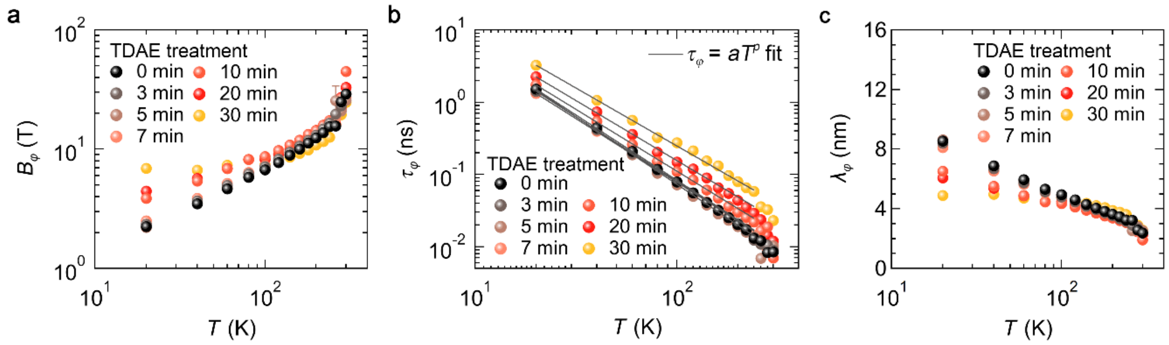

Figure S10. Inelastic scattering characteristics of the PEDOT:PSS–TFSA films as a function of TDAE treatment time. Temperature-dependent a) characteristic field B_φ, b) phase-breaking time τ_φ, and c) inelastic scattering length λ_φ. The values of B_φ were extracted by fitting the differential conductance ΔG versus B of the PEDOT:PSS films using 2D HLN model. The scattering parameter p was determined by interpreting the T dependency of the phase-breaking time τ_φ.

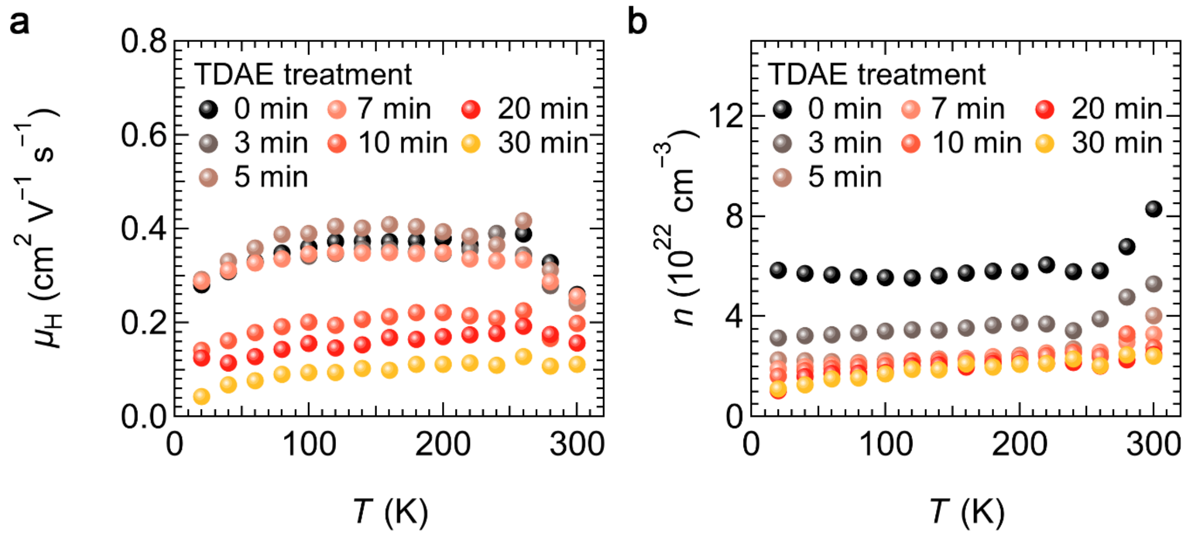

Figure S11. Electrical parameters of the TDAE-treated PEDOT:PSS–TFSA films extracted from Hall effect measurement. a) Temperature-dependent electrical mobility μ_H and b) carrier concentration *n*.

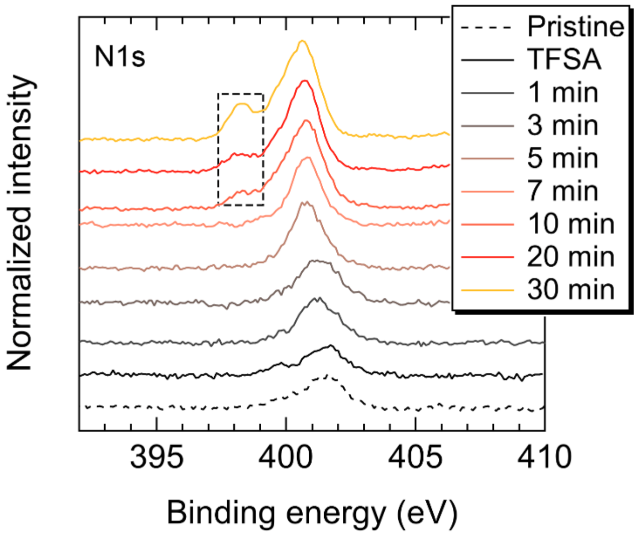

Figure S12. N1s XPS spectra of the pristine and PEDOT:PSS–TFSA films with different TDAE treatment time. The peaks at ~401.6 eV observed for the pristine and PEDOT:PSS–TFSA film correspond to the the protonated amines. As the TDAE treatment time increases, the peaks shifted to lower binding energy. When the TDAE treatment time exceeds 7 min, the peaks at 398.3 eV, which corresponds to the neutral amines, abruptly increases (marked by dotted rectangular).

Table S1. Information on the diffraction peak parameters derived from GIWAXS data. The values of crystal coherence length were calculated using the Scherrer formula.

Films	De-doping time	Crystallographic parameters of q_z (100)			Crystallographic parameters of q_{xy} (010)		
		q (Å^{-1})	d-spacing (Å)	Crystal coherence length (Å)	q (Å^{-1})	d-spacing (Å)	Crystal coherence length (Å)
Pristine	-	0.27	22.50	35.56	1.75	3.59	16.44
PEDOT:PSS –TFSA	0 min	0.47	13.34	52.35	1.82	3.44	38.98
	5 min	0.48	13.21	49.38	1.83	3.43	39.22
	7 min	0.46	13.66	49.61	1.84	3.42	37.49
	10 min	0.45	14.07	44.19	1.84	3.42	37.23
	30 min	0.43	14.46	41.44	1.82	3.44	37.66

Table S2. SLoT modelling and material parameters for the $\eta(c)$ and $W_H(c)$ relationships.

**Films**	c_{max}	A_0	A_1	$W_{H,max}$ **(meV)**	$W_{H,slope}$ **(meV)**	M_0 **(g mol^{-1})**	N	P **(g cm^{-1})**	k_BT **(meV)**
PEDOT:PSS–TFSA	0.5	0.2	6	710	1200	142	1.0	1.0	25.88

Table S3. Calculated σ_0, T_0, and T_1 of PEDOT:PSS–TFSA films as a function of TDAE de-doping time.

Films	De-doping time (min)	σ_0 (S cm^{-1})	T_0 (K)	T_1 (K)
PEDOT:PSS–TFSA	0	4150.5 (± 27.6)	81.8 (± 4.4)	46.9 (± 2.5)
	3	2503.3 (± 16.0)	72.3 (± 3.5)	50.6 (± 2.3)
	5	1836.3 (± 15.8)	59.4 (± 4.3)	43.9 (± 2.8)
	7	1593.3 (± 12.2)	52.7 (± 3.4)	43.7 (± 2.3)
	10	1130.1 (± 9.4)	55.3 (± 2.4)	83.8 (± 2.8)
	20	852.4 (± 9.7)	50.4 (± 2.7)	98.3 (± 3.8)
	30	629.3 (± 9.1)	42.4 (± 2.7)	128.4 (± 4.9)

References for Supporting Information

[1] S. D. Kang, G. J. Snyder, *Nat. Mater.* **2017**, *16*, 252.

[2] K. Kang, S. Schott, D. Venkateshvaran, K. Broch, G. Schweicher, D. Harkin, C. Jellett, C. B. Nielsen, I. McCulloch, H. Sirringhaus, *Mater. Today Phys.* **2019**, *8*, 112.

[3] S. A. Gregory, R. Hanus, A. Atassi, J. M. Rinehart, J. P. Wooding, A. K. Menon, M. D. Losego, G. J. Snyder, S. K. Yee, *Nat. Mater.* **2021**, *20*, 1414.

[4] D. Kim, Y. Park, D. Ju, G. Lee, W. Kwon, K. Cho, *Chem. Mater.* **2021**, *33*, 4853.

[5] J. L. Blackburn, S. D. Kang, M. J. Roos, B. Norton-Baker, E. M. Miller, A. J. Ferguson, *Adv. Electron. Mater.* **2019**, *5*, 1800910.

[6] A. B. Kaiser, *Adv. Mater.* **2001**, *13*, 927.

[7] K. Kang, S. Watanabe, K. Broch, A. Sepe, A. Brown, I. Nasrallah, M. Nikolka, Z. Fei, M. Heeney, D. Matsumoto, K. Marumoto, H. Tanaka, S.-i. Kuroda, H. Sirringhaus, *Nat. Mater.* **2016**, *15*, 896.

[8] Z. Fan, J. Ouyang, *Adv. Electron. Mater.* **2019**, *5*, 1800769.
